# Outcomes of belatacept-based quadruple immunosuppression therapy in kidney transplant recipients with persistent alloimmune response: a single-center observational study

**DOI:** 10.3389/fimmu.2026.1844666

**Published:** 2026-06-18

**Authors:** Basheer Ahamad Kummangal, Ali Olyaei, Angelo de Mattos, Shehzad Rehman, Justin Smith, Megan Stack, Zoha Ahmad, Jay Pandav

**Affiliations:** Division of Nephrology and Hypertension, Oregon Health and Science University, Portland, OR, United States

**Keywords:** belatacept, donor specific antibodies, immunosuppression, kidney transplantation, rejection

## Abstract

We conducted a retrospective review of adult kidney transplant recipients (KTRs) who received belatacept in addition to calcineurin inhibitors, antimetabolites, and corticosteroids for recurrent or refractory rejection or for persistent donor-specific antibodies (DSAs) unresponsive to standard therapy. Fifteen recipients were included. Kidney function remained stable on follow-up; median eGFR was 52 mL/min (R 23–91) at baseline and 54 mL/min (R 25–105) at 6 months (*p* = 0.15) after belatacept initiation. The DSA intensity declined significantly from a median of 4,990 mean fluorescence intensity (MFI) (R 0–23,305) at baseline to 1,644 MFI (R 0–6,903) at 3–6 months (*p* = 0.03). There was also a small reduction in biopsy-proven rejections from 85.7% to 63.6%. Infections occurred in most recipients (73.3%), with a majority arising more than 6 months after therapy initiation. Two patients developed post-transplant lymphoproliferative disorder (PTLD), each with either substantial prior exposure to lymphocyte-depleting agents or prolonged immunosuppression. Two deaths occurred, one related to PTLD and one to septic shock. These findings suggest that belatacept-based quadruple immunosuppression may reduce DSA and stabilize kidney function in patients with persistent alloimmunity, but without a statistically significant reduction in rejections. There is also a significant burden of infections and PTLD, highlighting the need for careful patient selection and caution before adopting this approach.

## Introduction

Belatacept, a high-affinity variant of the CTLA4-Ig fusion protein with two amino acid substitutions (L104E and A29Y) that increase its avidity for B7, blocks CD28-mediated T-cell costimulation (1). It was developed as a calcineurin inhibitor (CNI) alternative to avoid nephrotoxicity and preserve long-term kidney function (2). The BENEFIT trial conducted in standard criteria donor kidney recipients showed that *de novo* belatacept had a lower risk of death or graft loss, improved renal function, and lower donor-specific antibody (DSA) compared to CNI-based therapy (3). The BENEFIT-EXT trial similarly demonstrated improved renal function with belatacept in extended criteria donor kidney recipients (4). This improvement in renal function has also been reported after conversion from CNI to belatacept (5). However, *de novo* belatacept has been associated with an increased risk of acute cellular rejection, particularly early after transplantation (3). This risk is reduced with a short course of CNI before belatacept initiation (6). The increased rejection risk is multifactorial. One proposed mechanism is the downregulation of CD28 on memory T-cell membranes under belatacept treatment (7). However, newer evidence suggests that CD28+ memory T cells may themselves be mediators of belatacept-resistant rejection, retaining proliferative capacity despite belatacept exposure before eventually losing CD28 expression and infiltrating the allograft as terminally differentiated effectors (8). Early rejection risk could also be increased by induction therapy with lymphodepleting agents, which may enrich depletion-resistant memory T cells (9–12). Belatacept therapy is associated with increased risk of post-transplant lymphoproliferative disorder (PTLD), especially in Epstein–Barr Virus (EBV)-seronegative recipients. Hence, its use is restricted to EBV-seropositive patients (13). Reported infection outcomes have been mixed. Post-conversion and CMV high-risk populations have described an increased number, severity, and atypical presentations of opportunistic infections, especially CMV (14–17). BENEFIT and BENEFIT-EXT trials reported similar rates of infection in *de novo* belatacept and CNI groups (3, 4).

Belatacept provides signal−2 blockade of T−cell co−stimulation, which may be synergistic with the downstream signal−1 blockade achieved by CNI (18). This complementary immunosuppressive effect may provide a potential treatment strategy in kidney transplant recipients (KTRs) with a persistent alloimmune response. In combination, CNI can potentially blunt the risk of rejection, and belatacept can reduce the formation of DSA, while retaining the favorable metabolic and GFR-preserving effects of belatacept (3, 6, 19). An analysis of the 2010–2022 UNOS STAR database reported that belatacept plus tacrolimus was associated with lower risks of death and all-cause graft failure than tacrolimus alone, with more pronounced benefits among older recipients (20). A preliminary series evaluating quadruple immunosuppression (QIS) therapy with belatacept, CNI, antimetabolite, and corticosteroids for refractory antibody-mediated rejection observed reductions in DSA levels, but persistence of rejection, and more severe infections (21).

## Materials and methods

We performed a single-center retrospective chart review of all adult KTRs who were started on belatacept-based QIS for persistent alloimmune response at Oregon Health and Science University. The study was approved by the Oregon Health and Science University Institutional Review Board (IRB Number 28949), and all procedures were performed in compliance with relevant laws and institutional guidelines. Given the retrospective design and minimal risk to patients, requirement for informed consent was waived by the IRB. Belatacept-based QIS consisted of belatacept added to the triple regimen of CNI, antimetabolites, and corticosteroids. Belatacept was administered intravenously at a dose of 5 mg/kg every 2 weeks for five doses and then every 4 weeks thereafter. The standard CNI used was tacrolimus, titrated to a target trough level of 5–10 ng/mL. In case of tacrolimus intolerance, cyclosporine was used with a trough target of 100–150 ng/mL. Maintenance corticosteroid therapy consisted of prednisone at a daily dose of 5–10 mg. The antimetabolite could be either mycophenolate mofetil 1,000 mg twice daily, mycophenolate sodium 720 mg twice daily, or, if these were not tolerated, azathioprine 1–2 mg/kg daily. Persistent alloimmune response was defined as recurrent or refractory biopsy-proven rejection, or persistent DSA not improving with standard therapy. This consisted of maximizing maintenance immunosuppression (CNI: tacrolimus trough target 8–12 ng/mL or cyclosporine trough target 150–200 ng/mL, antimetabolite: mycophenolate mofetil 2.5–3 g per day or azathioprine 2–3 mg/kg/day, prednisone 5–10 mg per day). If mean fluorescence intensity (MFI) was higher than 10,000, rituximab was considered. Our five patients who had persistent DSA as the indication for starting QIS were previously treated with maximizing triple therapy, with one patient also receiving rituximab. Patients who were converted to belatacept from CNI due to its adverse effects or intolerance were excluded. All EBV-seronegative recipients were also excluded. Outcomes of interest were eGFR trend, total DSA MFI trend, biopsy-proven rejections, infections, malignancies, patient, and graft survival. Continuous paired outcomes (eGFR and DSA MFI) were analyzed using the Wilcoxon signed rank test. Categorical variables were summarized using counts and proportions. Descriptive data were reported as medians with ranges. Analyses were performed using SPSS version 30.

## Results

Fifteen KTRs fulfilled the inclusion criteria and were included in the study (**Table 1**). Out of these, 13 were white patients (86.7%) and two were Hispanic patients (13.3%). Eight KTRs were female (53.3%), and seven were male (46.7%). Median age was 46 years (R 20–64). Median transplant vintage at QIS initiation was 3.0 years (R 0.2–24.2). Median follow-up after QIS was 1.3 years (R 0.3–1.7). Twelve of these were kidney-alone transplants (80.0%) while three were simultaneous kidney pancreas (SPK) transplants (20.0%). Three were living donor kidney transplants (20.0%), while 12 were deceased donor kidney transplants (80.0%). Ten patients (66.7%) were first-time transplant recipients. Three patients (20.0%) had one previous kidney transplant, while one had two previous kidney transplants and one patient had a previous liver transplant (6.7% each). Diabetic nephropathy and immune-mediated glomerulonephritis were the most common native kidney disease, each at 4 (4/15, 26.7% each). Median cPRA was 5% (R 0–100), with a median human leukocyte antigen (HLA) mismatch of 4 (R 0–6). Median kidney donor profile index (KDPI) of deceased donors was 18 (R 1–63). Median CIT was 19.2 h (R 1.0-26.7 h). Fourteen KTRs (93.3%) received induction with a lymphodepleting agent. Out of these, 11 received antithymocyte globulin (ATG) 1.5 mg/kg daily over 3–4 days, and 3 received a 30-mg dose of alemtuzumab. One KTR (6.7%) received non-lymphodepleting induction with basiliximab 20 mg on post-operative days 0 and 3. Fourteen patients (93.3%) were on tacrolimus, while one (6.7%) was on cyclosporine, in addition to prednisone, antimetabolite, and belatacept. Five KTRs developed delayed graft function (33.3%), all of which subsequently resolved. Nine (60%) had intermediate-risk CMV status defined as donor positive, recipient positive, or donor negative, recipient positive combination, while three had high risk defined as donor positive, recipient negative combination, and three had low risk defined as donor and recipient negative combination (20.0% each). Indication for QIS initiation was recurrent or refractory rejection in 10 KTRs (66.7%) and persistent DSA in 5 (33.3%). Among the five patients with persistent DSA as the indication, two also had the first episode of rejection in biopsy. Two KTRs did not have any rejection in the pre-QIS biopsy, and one did not have any biopsy performed. Among the total 12 rejections (12/14, 85.7%), 7 had ABMR (58.3%), 3 had TCMR (25%), and 2 had mixed rejection (16.7%). Three of the 10 (30%) KTRs with recurrent or refractory rejection had a positive flow cytometry crossmatch, while none of the patients in the persistent DSA cohort had a DSA barrier at the time of transplantation. Nonadherence was a concern in two patients with ABMR prior to initiation of QIS, but no adherence issues were identified during QIS therapy. The median duration of QIS was 323 days (R 68–470 days). The reason for cessation of QIS was infection (4/15, 26.7%), CNI intolerance (3/15, 20.0%), and malignancy (2/15, 13.3%). Three KTRs (20.0%) were electively weaned off after approximately 6–12 months of QIS without any intolerance and three KTRs (20.0%) had ongoing QIS at the time of analysis.

**Table 1:** Patient demographics, transplant characteristics, rejection history, and Banff classification before and during Quadruple Immunosuppression (QIS).

Pt	Age	Sex	Native renal disease	Organ/Donor/Tx #	Induction	Prior BPAR (total - type x n)	Prior rejection treatment (in addition to pulse steroids)	History of DSA (class × episodes)	Cross-match history	Banff at biopsy leading to QIS	Banff on QIS (follow-up biopsy)
1	34	M	Reflux nephropathy	Kidney/LRRT/1st	Basiliximab	1 - ABMR ×1	Rituximab ×2; IVIG ×1	Class II ×1	Negative	Chronic active ABMR	Chronic active ABMR
2	20	F	Prenatal cortical necrosis	Kidney/DBD/2nd	Alemtuzumab	3 - Mixed ×2; ABMR ×1	ATG ×1; Rituximab ×2; IVIG ×1; Tocilizumab	Class II ×8	Negative	Chronic active ABMR + chronic active TCMR Grade 1A	Chronic active ABMR
3	47	F	Reflux nephropathy	Kidney/DBD/2nd	Thymoglobulin	7 - ACR ×4; Mixed ×2; ABMR ×1	ATG ×1; IVIG ×2; PP ×2	Class I + II ×11	Positive B-cell flow XM	Chronic active TCMR Grade IA	Chronic active TCMR Grade IA
4	51	M	Type 1 DM	SPK/DBD/1st	Alemtuzumab	3 - ABMR ×3	Rituximab ×2; IVIG ×3; PP ×3; Bortezomib ×1	Class I + II ×7	Negative	No biopsy	Chronic and active ABMR
5	52	M	SLE + congenital single kidney	Kidney/LURT/1st	Thymoglobulin	1 - ABMR ×1	Rituximab ×2; IVIG ×2; PP ×2	Class II ×1	Negative	Chronic active ABMR	-
6	62	F	IgA nephropathy	Kidney/DCD/1st	Thymoglobulin	1 - ABMR ×1	IVIG ×1	Class I ×8; Class II ×1	Positive B & T cell flow XM	Active ABMR	Active ABMR
7	45	M	Hepatorenal syndrome	Kidney/DBD/2nd (prev liver)	Thymoglobulin	1 - ABMR ×1	Rituximab ×1	Class II ×2	Negative	Active ABMR	No rejection
8	46	M	Type 1 DM	SPK/DBD/1st	Thymoglobulin	1 - ACR ×1	Pulse steroids only	None	Negative	Chronic active TCMR Banff 1B	-
9	34	F	Type 1 DM	Kidney/DBD/1st	Thymoglobulin	3 - ABMR ×3	Rituximab ×2; IVIG ×1; PP ×1	None	Negative	Active ABMR	-
10	55	F	SLE	Kidney/DBD/2nd	Thymoglobulin	0	-	Class II ×2	Negative	No rejection	No rejection
11	64	F	Renal agenesis/dysgenesis	Kidney/DCD/1st	Thymoglobulin	1 - ACR ×1	ATG ×1	None	Negative	Acute TCMR Grade 1B	No rejection
12	37	M	Cystinosis	Kidney/DBD/3rd	Thymoglobulin	1 - ABMR ×1	Rituximab ×1; IVIG ×2; PP ×1	Class I ×2	Positive B & T cell flow XM	Active ABMR	Active ABMR + acute TCMR Grade 1A
13	48	F	IgA nephropathy	Kidney/DCD/1st	Thymoglobulin	0	-	Class I + II ×8	Negative	No rejection	No rejection
14	42	F	Type 1 DM	SPK/DBD/1st	Thymoglobulin	0	-	Class I ×1; Class II ×2	Negative	Borderline TCMR + microvascular inflammation	-
15	21	M	Renal hypoplasia/dysplasia	Kidney/LRRT/1st	Alemtuzumab	3 - ACR ×1; ABMR ×2	Rituximab ×2; IVIG ×2; PP ×1	Class II ×3	Negative	Active ABMR	Chronic active ABMR

Tx#, transplant number; LRRT, living related renal transplant; LURT, living unrelated renal transplant; DBD, donation after brain death; DCD, donation after circulatory death; SPK, simultaneous pancreas kidney. BPAR, biopsy proven acute rejection. ABMR, antibody mediated rejection; TCMR, T cell mediated rejection.

**Figure 1:**
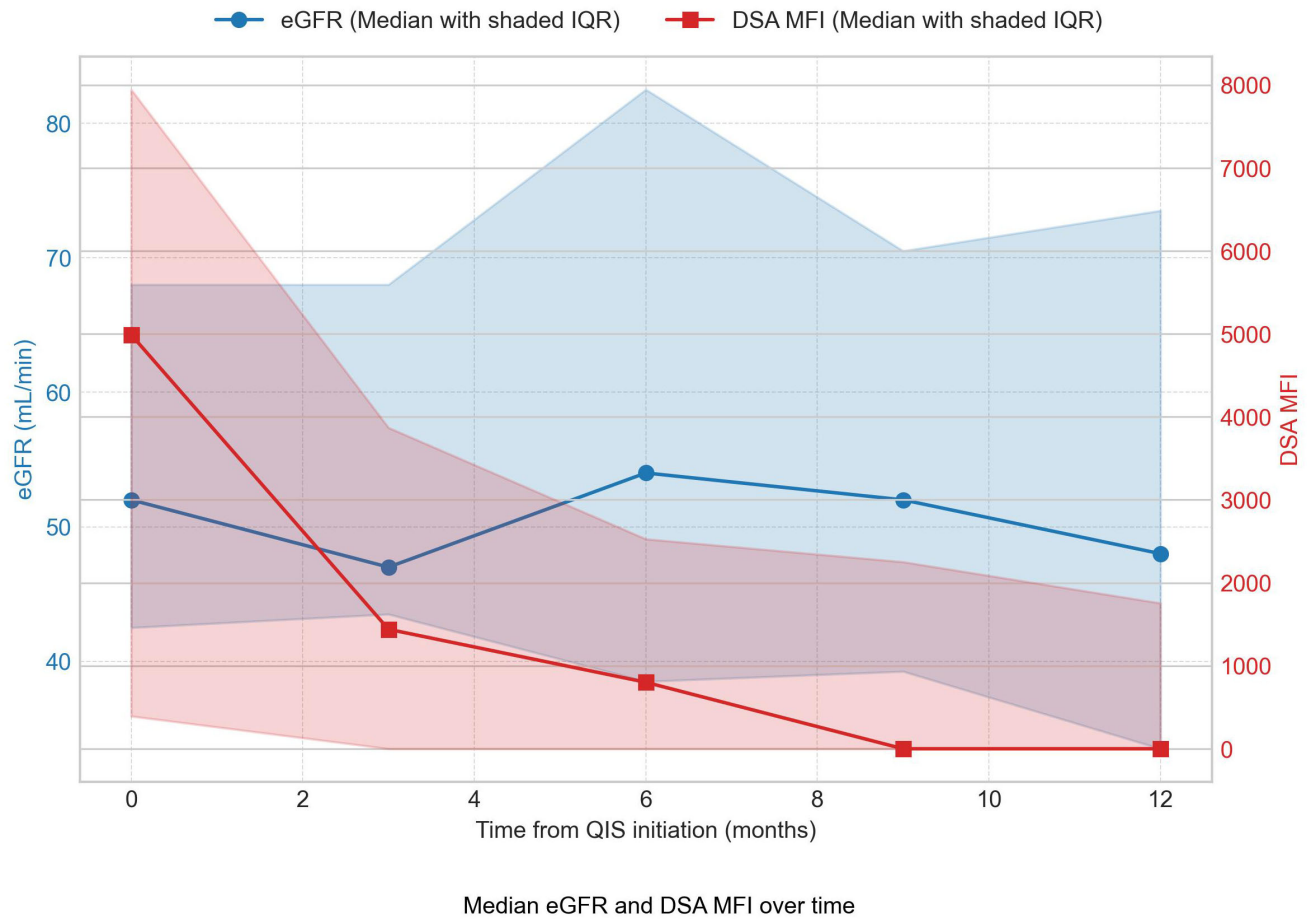
Trend of eGFR and DSA MFI following belatacept-based quadruple immunosuppression (QIS). DSA, donor-specific antibody; MFI, mean fluorescence intensity.

At QIS initiation, median eGFR was 52 mL/min (R 23–91). At follow-up in 6 months, eGFR was 54 mL/min (R 25–105). This was not statistically different by the Wilcoxon signed rank test (*p* = 0.15). Subgroup analysis by indication further confirmed this stability. In the persistent DSA group, the median eGFR was 63 (R 33–91) at initiation and 68 (R 25–105) at 6 months (*p* = 0.35), while the rejection group trended from 50 (R 23–76) to 53.5 (R 35–102, *p* = 0.28). Median DSA MFI trended down from 4,990 MFI (R 0–23,305) before QIS to 1,644 MFI (R 0–6,903) at 3–6 months following QIS (**Figure 1**). This was statistically significant by the Wilcoxon signed rank test (*p* = 0.03). Follow-up biopsies were performed in 11 KTRs, and 7 (63.6%) continued to show rejection, compared to 12/14 (85.7%) before QIS. Among the persistent rejections, five had ABMR (71.4%), while one each had TCMR and mixed rejection (14.3% each). Eleven KTRs developed infections during follow-up (73.3%). There was a total of 24 infections in these 11 patients, resulting in 15 episodes of hospitalization. Most of the infections occurred more than 6 months after the initiation of the quadruple immunosuppression (18/24, 75%). Half of the infections were bacterial (12/24, 50%), followed closely by viral infections (10/24, 41.7%). The most common site of infection was the respiratory tract (11/24, 45.8%) (**Table 2**). There were three episodes of CMV infection (3/24, 12.5%) in three patients (3/15, 20%), including one case of CMV pneumonitis and two cases of CMV viremia. There was one episode of BK viremia and no EBV infection. Two patients developed malignancies, both of which were PTLD (13.3%). One of them had a cumulative ATG dose of 1,075 mg for recurrent T cell-mediated rejection and a cumulative immunosuppression vintage of 1.9 years at PTLD diagnosis. The other KTR had only one dose of lymphodepleting agent (alemtuzumab 30 mg) but a total immunosuppression vintage of 9.6 years. Two graft failures occurred, one at 394 days after QIS initiation due to acute illness and contrast exposure as a result of PTLD complications. This KTR died at 424 days post QIS initiation due to PTLD. Another KTR died at 512 days due to septic shock with a functioning graft. Both of these KTRs were on QIS till the diagnosis of their terminal condition.

**Table 2:** Summary of infections following belatacept-based quadruple immunosuppression (QIS).

Outcomes	Number	Percentage
Patient level outcomes (n = 15)		
Patients with ≥ 1 infection	11	73.3%
Infection level outcomes (n = 24)		
Infections ≤ 6 months after QIS initiation	6	25.0%
Bacterial infections	12	50.0%
Viral infections	10	41.7%
Fungal infections	2	8.3%
Pulmonary infections	11	45.8%
Bloodstream infections	4	16.7%
Gastrointestinal infections	3	12.5%
Genitourinary infections	3	12.5%
Skin and soft tissue infections	3	12.5%
Hospitalizations		
Number of infection-related hospitalizations	15	

## Discussion

This study assesses the outcomes after the off-label addition of belatacept to the traditional triple immunosuppression for KTRs with persistent alloimmune response. The renal function was stable during follow-up. This is in line with previous studies, which showed that belatacept alone or in combination with CNI was superior to CNI alone in preserving renal function (3, 4, 19, 20). This renal function preservation may reflect reduced CNI nephrotoxicity and suppression of DSA (22). The DSA MFI showed a statistically and clinically significant improvement. This correlates with the consistent suppression of DSA by belatacept that has been demonstrated consistently across multiple studies (23). This might be achieved through a shift in the B-cell compartment toward naïve and transitional phenotypes associated with immune regulation and tolerance, and downregulation of B-cell activating factor (BAFF) pathways limiting B-cell activation (24).

Although rejection persisted in the majority of follow-up biopsies, there was a slight decrease from 85.7% to 63.6%. This reduction might be related to the suppression of DSA. However, it is less impressive compared to the improvement in DSA. This finding is consistent with previous registry studies, which showed that a combination of CNI and belatacept immediately after transplant displayed similar or slightly worse incidence of rejection compared to CNI alone, but better than belatacept alone (19, 20). This is likely due to the proliferation of CD28− memory T cells as well as CD28+ memory T cells that retain proliferative and effector function in the presence of belatacept (7, 8). These can escape costimulatory blockade and lead to rejection despite the suppression of DSA.

Infections developed in 73.3% (11/15) of the KTRs. Of these 11, 10 (91%) had received a lymphodepleting induction regimen (8 with ATG and 2 with alemtuzumab) and 1 patient (9%) received ATG again for treatment of rejection. This infection burden is similar to the 70% noted in the BENEFIT trial in the comparable less intense (LI) belatacept regimen arm and 71% noted in the CNI arm at a similar follow-up duration (25). Thus, even though the infection burden is significant, the combination of belatacept and CNI might not lead to a synergistic increase in the infection risk. However, 20% of the KTRs developed CMV infection, which is higher than noted in the BENEFIT trial (3%–4%) (25) and retrospective studies (14, 17). Adams et al., who studied *de novo* belatacept with and without tacrolimus, noted that the CMV viremia was higher in the cohort who received belatacept only, which was suspected to be due to the increased number of rejections and subsequent exposure to higher immunosuppression (6). Similarly, the higher rates of infection in our study might be at least partly due to the previous alloimmunity treatment. Most of the infections occurred after 6 months of the start of QIS. This delayed increase in the infection burden is similar to a study on opportunistic infections in KTRs after belatacept conversion (14). This delay might reflect a progressive accumulation of immunosuppression burden.

Two patients developed PTLD on follow-up. This represented 13.3% of the total cohort. They had either a high cumulative dose of ATG exposure or a long duration of immunosuppression exposure. A large retrospective observational study on KTRs over the past three decades has shown an incidence of approximately 1.5% (26). Our patients, by virtue of persistent alloimmunity, have been exposed to higher doses of immunosuppression, including high doses of ATG. Multiple studies have revealed ATG as a risk factor for the development of PTLD (27–30). The duration of immunosuppression is also likely a contributor, as a registry study from Australia and New Zealand has shown a reduction in PTLD incidence after graft failure and cessation of immunosuppression (31). It is unclear how much of the increased risk of PTLD is due to belatacept. Belatacept has been found to increase the risk of PTLD in EBV-seronegative KTRs (13). However, this patient population was excluded from our study. National OPTN data have not shown an increase in PTLD with belatacept in EBV-seropositive patients (32). In addition to the exposure to rejection and DSA treatments, our cohort was also exposed to more intensive induction and higher targets of CNI compared to the *de novo* belatacept study by Adams et al. (6) A lower-intensity immunosuppression accompanying belatacept might have lowered the infection and PTLD burden.

This study has several important limitations. The sample size is small and lacks adequate power to detect significant differences in the rates of rejection before and after the QIS. Another key limitation is the absence of a control group. It is unclear how the stability in eGFR observed in our QIS cohort compares with that of patients maintained on conventional triple immunosuppression. Similarly, the incidence of infectious complications in this group is difficult to contextualize without a control group receiving standard triple therapy, particularly given that both groups would likely be exposed to intensified immunosuppression for the treatment of DSA or rejection. The heterogeneity in the immunosuppression regimen before and during the QIS, including the induction regimen and the type of CNI, limits the interpretation of the data. This variability is further compounded by the diverse indications for initiating QIS therapy. The patient population lacked African American and Asian representation, affecting its generalizability. There was an absence of protocolized biopsies and long-term follow-up. Metabolic effects of QIS were also not studied. Belatacept is not currently approved for use in SPK transplantation; however, 20% of our cohort consisted of SPK recipients.

This study demonstrates that belatacept-based QIS is associated with significant reductions in DSA and stable kidney function in patients with persistent alloimmunity, but these immunological benefits were not accompanied by a statistically significant reduction in rejections. This high-risk patient population also suffered a high burden of infectious complications and PTLD, demonstrating the need for cautious patient selection before adopting this strategy. Larger controlled studies with clearly defined patient selection criteria and structured infection and PTLD surveillance are required to precisely assess the benefits as well as the risk of infections and PTLD with belatacept-based QIS compared to conventional immunosuppression in this population.

## Data Availability

The raw data supporting the conclusions of this article will be made available by the authors, without undue reservation.
